# Enhancing Health Equity by Predicting Missed Appointments in Health Care: Machine Learning Study

**DOI:** 10.2196/48273

**Published:** 2024-01-12

**Authors:** Yi Yang, Samaneh Madanian, David Parry

**Affiliations:** 1 Auckland University of Technology Auckland New Zealand; 2 Murdoch University Perth Australia

**Keywords:** Did Not Show, Did Not Attend, machine learning, prediction, decision support system, health care operation, data analytics, patients no-show, predictive modeling, appointment nonadherence, health equity

## Abstract

**Background:**

The phenomenon of patients missing booked appointments without canceling them—known as Did Not Show (DNS), Did Not Attend (DNA), or Failed To Attend (FTA)—has a detrimental effect on patients’ health and results in massive health care resource wastage.

**Objective:**

Our objective was to develop machine learning (ML) models and evaluate their performance in predicting the likelihood of DNS for hospital outpatient appointments at the MidCentral District Health Board (MDHB) in New Zealand.

**Methods:**

We sourced 5 years of MDHB outpatient records (a total of 1,080,566 outpatient visits) to build the ML prediction models. We developed 3 ML models using logistic regression, random forest, and Extreme Gradient Boosting (XGBoost). Subsequently, 10-fold cross-validation and hyperparameter tuning were deployed to minimize model bias and boost the algorithms’ prediction strength. All models were evaluated against accuracy, sensitivity, specificity, and area under the receiver operating characteristic (AUROC) curve metrics.

**Results:**

Based on 5 years of MDHB data, the best prediction classifier was XGBoost, with an area under the curve (AUC) of 0.92, sensitivity of 0.83, and specificity of 0.85. The patients’ DNS history, age, ethnicity, and appointment lead time significantly contributed to DNS prediction. An ML system trained on a large data set can produce useful levels of DNS prediction.

**Conclusions:**

This research is one of the very first published studies that use ML technologies to assist with DNS management in New Zealand. It is a proof of concept and could be used to benchmark DNS predictions for the MDHB and other district health boards. We encourage conducting additional qualitative research to investigate the root cause of DNS issues and potential solutions. Addressing DNS using better strategies potentially can result in better utilization of health care resources and improve health equity.

## Introduction

Adding to the existing pressures on the health care system [1,2], further substantial disruptions are caused when patients fail to attend their prescheduled appointments [3]. This is defined as Did Not Show (DNS), which is a scheduled but not utilized clinical appointment that patients failed to attend without canceling or rescheduling. This phenomenon is also known as Did Not Attend (DNA) or Failed To Attend (FTA). Causes include the patient forgetting about their appointment, miscommunication [4], logistical difficulties, appointment scheduling conflicts, and family/work commitments [3,5].

DNS can adversely affect patients’ well-being, cause them and the system financial stress, and disturb health care operations and systems. Globally, DNS has an overall rate of 23%, with a wide geographical variation (13.2% in Oceania, 19.3% in Europe, 23.5% in North America, 27.8% in Asia, and 43% in South America [6]). DNS is expensive for health systems; for example, estimated annual losses amounting to £790 million (over US $1 billion) were found in the United Kingdom [7] and $564 million in the United States [8]. It affects both primary and secondary health care [9], although secondary care losses are higher.

Patients mostly fail to comply with their clinical appointments when symptoms become less severe or unnoticeable [10,11], which might deteriorate underlying syndromes [12,13]. Patients are more likely to demand immediate medical attention when contracting serious health issues or require acute and emergency care if they miss scheduled health care appointments [12,14-16].

Eliminating DNS is hard to achieve, and its adverse effects necessitate methods and approaches for managing DNS such as sending digital reminders by text, phone, and email [17,18]. These approaches have not been very effective, as they are time-consuming and costly, and the health care system still faces DNS issues. Overbooking [3,19], open access [20], and DNS penalty approaches have also been used to enhance clinical slot utilization but can cause longer waiting times for patients and overtime for clinical staff [21].

Inspired by the success of artificial intelligence (AI) in different sectors, including health care [22,23], we considered the application of AI for DNS management via predicting the probability of DNS appointments [13,19,24,25]. AI and its subset techniques, such as machine learning (ML), are powerful for extracting cognitive insights from massive amounts of data [26,27].

The predicted DNS probabilities proved to be successful in providing the required information for DNS management [25] and supporting health care managers in making informed decisions for prioritizing patients and delivering clinical assistance. This enables health care providers to reschedule and reuse limited clinical resources for urgent cases while also expanding access to health care services for patients from diverse backgrounds, thereby promoting health care equity.

Therefore, clinical capabilities and medical resources can be used more effectively and efficiently, decreasing patients’ wait times, increasing their satisfaction, and enhancing health productivity.

Most studies concerned with predicting DNS have mainly comprised small data sets or specific groups of people to develop models for DNS learning and prediction; however, DNS tends to be varied across populations. For example, longer distances to a medical facility increase DNS [8], but this finding was contradicted in another study [28]. Likewise, patients with chronic illnesses adhere to their scheduled appointments [13], while other studies [29] have shown that patients with more severe diseases have a higher DNS rate. Even within a single medical organization, DNS factors vary across different clinics [14]. These examples highlight the inconsistent nature of DNS predictors, showcasing the complexity of predicting tasks in this domain. Such variations pose challenges in creating a universal formula or model to effectively address DNS prediction issues on a global scale.

Considering the very limited DNS research in New Zealand and the complexity of developing a general DNS predictive model, we concentrated on the DNS issue in the MidCentral District Health Board (MDHB) hospital as a proof of concept. MDHB is located in the center of the North Island, New Zealand, covering a land area of over 8912 km^2^ and with a population of over 191,100 people. In this region, about 18% of people are aged 65 years or older, with over 20% being Māori, and a higher proportion than the national average resides in more deprived areas [30]. These demographic factors could lead to inequity in access to health care services. To support MDHB in addressing health equity and providing additional support for patients, this study aimed to develop ML models and compare their performance in predicting the probabilities of future DNS appointments at MDHB. This study utilized a data set spanning 5 years of collected data.

## Methods

### Overview

**Figure 1:**
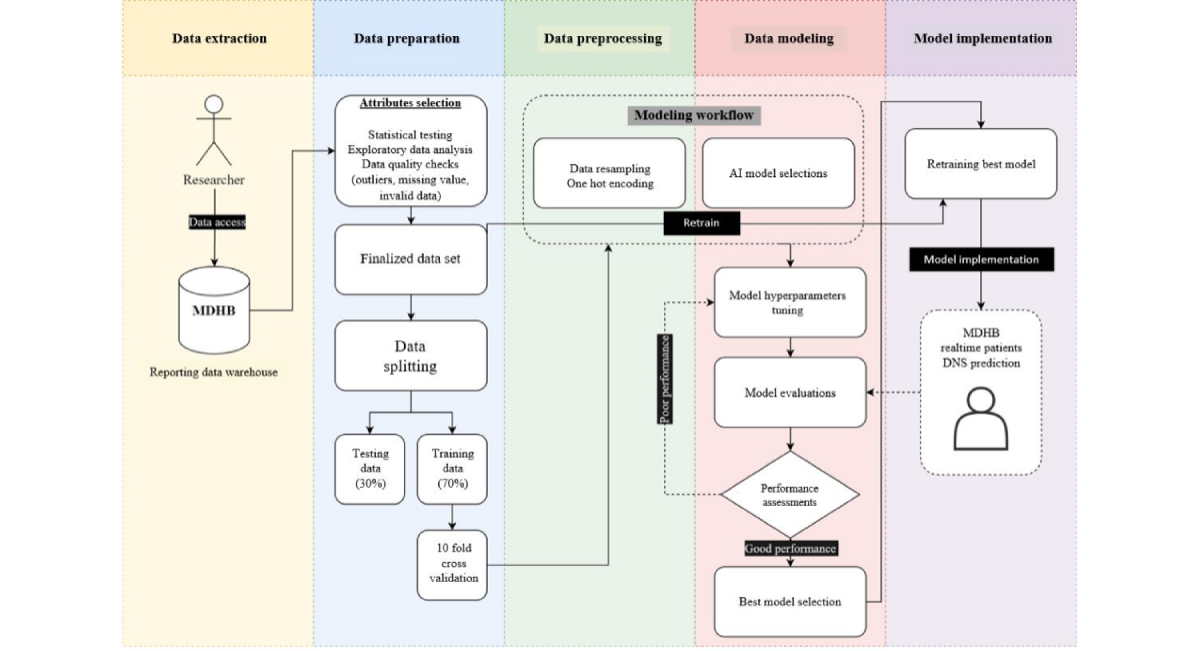
Research flow and procedure. AI: artificial intelligence; MDHB: MidCentral District Health Board.

Our research was organized into the following phases (Figure 1)**.** The initial phase involved *data extraction*, defining the data set to be used, and outlining the data extraction process. The *data preparation* phase involved conducting exploratory data analysis (EDA) to profile data and exclude irrelevant observations from the research. Subsequently, the data set was split into 2 parts—70% (454,831 records) for training and 30% (194,927 records) for testing. To avoid data linkage, the training and testing data sets were not mixed during the ML modeling phase. Moreover, the training set underwent a 10-fold cross-validation strategy to prevent bias as much as possible and fully utilize its limited training information. Next, the *data preprocessing* phase involved cleaning and transforming the cross-validation sets, ensuring that the training set was ready for the data modeling stage. A 10-fold cross-validation resampling strategy was applied to further optimize the utilization of the 70% training data. In the *data modeling* phase, we used 3 ML algorithms and tuned their hyperparameters to identify the best performance among the algorithms. Finally, in the *model evaluation* phase, various evaluation metrics were employed to determine the best-performing ML model for DNS prediction.

### Data Access and Extraction

**Figure 2:**
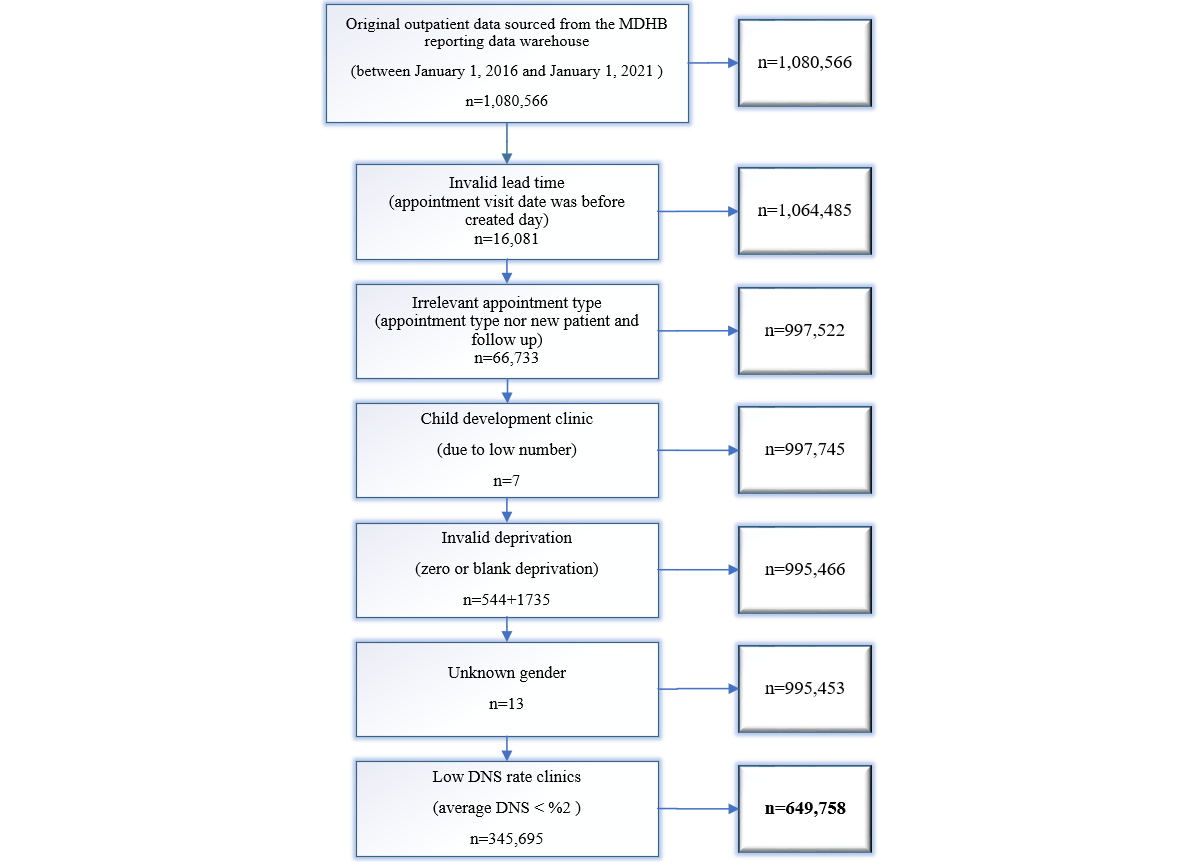
Research data exclusion. DNS: Did Not Show; MDHB: MidCentral District Health Board.

Our data were sourced from MDHB reporting SQL farm and contained only outpatient visits with no link to other data sets. This significantly mitigated risks related to patient reidentification. Data deidentification and encryption were applied before data access, and New Zealand National Health Index numbers were encrypted to protect patients’ privacy. We acquired 1,080,566 outpatient visit records from 38 clinics between January 1, 2016, and December 31, 2020, satisfying the research requirements with almost 57,000 DNS incidents (5% of the entire data set). The steps of data exclusion are presented in Figure 2. Because not many missing records were identified in the data sets, those with missed values were directly excluded.

### Ethical Considerations

This study received ethics approval from the Auckland University of Technology (AUT; 20/303) and MDHB (2020.008.003), following which data access to the MDHB reporting data warehouse was granted.

### Data Preparation

#### Phase Description

**Figure 3:**
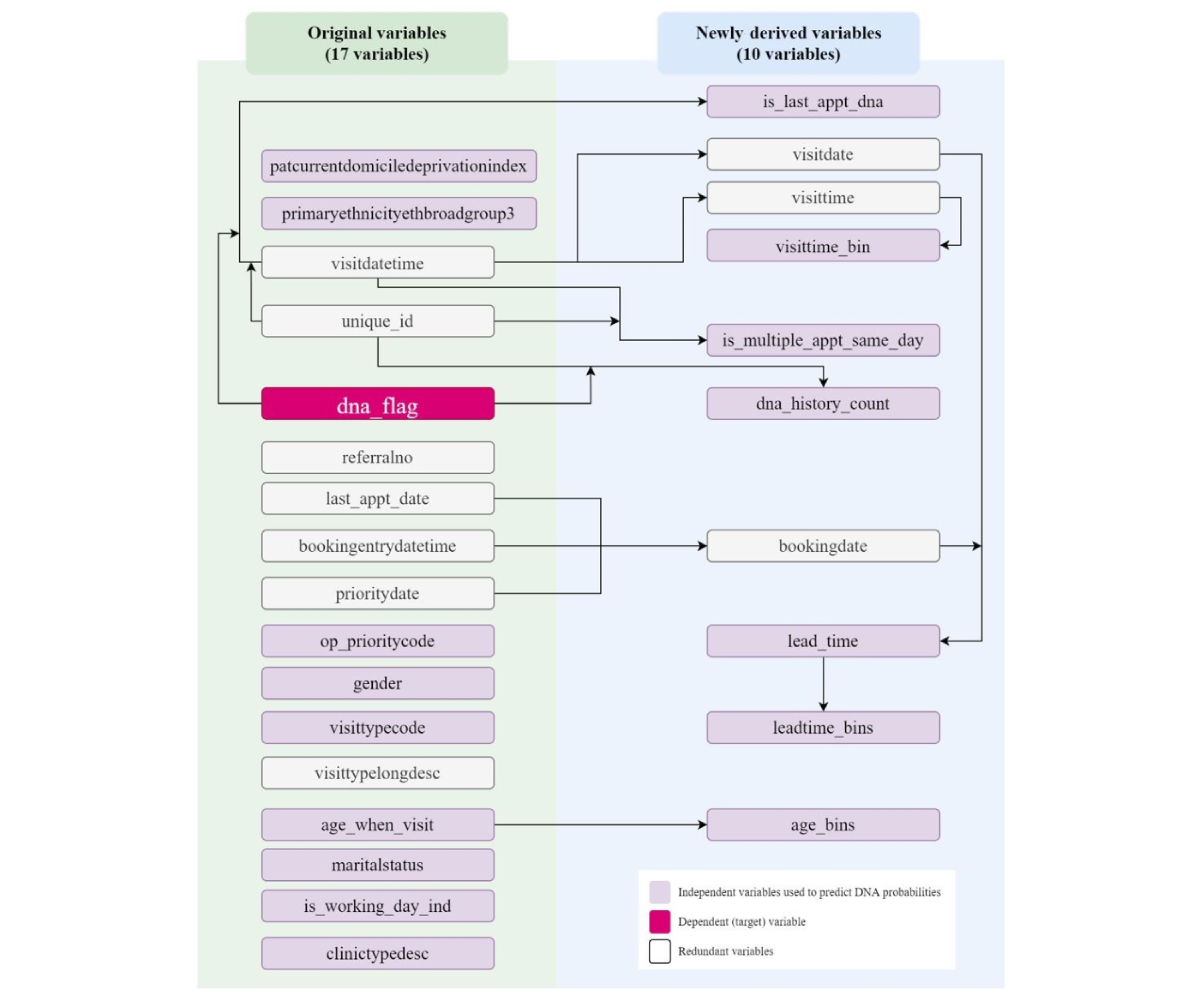
Variable transformation and derivation visualization.

In this phase, understanding the data was important to adequately prepare them for the experiments. The data preparation process included data transformation and derivation (Figure 3). Following suggestions from the literature, new research variables were derived and introduced because some valuable DNS predictors were absent in the MDHB data set. For example, no direct information was available on the patients’ DNS history [21,31], appointment lead time [31,32], or latest appointment DNS outcome [13]. The lead time was calculated by comparing the difference in days between the appointment creation date and the visit date. Appointments with longer lead times were expected to have greater DNA probability than those with shorter lead times [29].

Therefore, to better understand patient behavior and DNS patterns, we derived 10 new variables on top of the original variables (Figure 3). These attributes were introduced to support us in understanding when patients were more likely to miss their appointments in general and to identify regular nonadherent patients.

Initially, we extracted a data set with 17 columns and over 1 million records (Multimedia Appendix 1). Informed by the literature review [14,29,31-33], we derived and introduced another 10 variables on top of the original data and increased the data columns to 27. Among all the variables, 16 (59%) were used for ML modeling, and the redundant ones were excluded. The *dna_flag* attribute was the dependent (target) variable. Figure 3 demonstrates the original variables in addition to 10 newly derived ones.

#### Cardinality Reduction

We conducted a cardinality reduction analysis to reduce variable categories with low frequency and small samples. The data set mostly included categorical variables, with numeric variables being rare. Each categorical level is called a cardinality, which means how many distinct values are in a column. In our data set, some categorical variables had fewer levels, such as patient gender—M (male), F (female), and U (unknown)—while others had hundreds of variations, such as suburbs or diagnosis codes.

Developing ML models often involves numerous categorical attributes, necessitating examination of the variables’ cardinality, as most ML algorithms are distance-based and require converting categorical variables to numeric values. Categorical variables with high cardinality levels will derive massive new columns and expand the data set. This expansion increases model complexity, elevates computational costs, and decreases model generalization, which makes handling the data set challenging [34]. Therefore, we investigated the cardinality of our research variables and deployed a reduction strategy accordingly.

Cardinality reduction analysis was conducted to reduce the number of categories within variables with low frequency and small sample sizes. Following suggestions from the literature, new research variables were also derived and introduced, including patients’ prior DNS history [14,16,21] and the appointment lead time [14,16,29,32].

#### Statistical Test

The chi-square test was used for analyzing homogeneity among different groups within variables [35] and for testing the independence between categorical variables [36]. The chi-square statistics (*χ*^2^) and their *P* values were calculated to investigate whether different levels of a variable contributed differently to DNS events.

The confidence level (α=.05) was adopted as the *P* value threshold in the chi-square test. A *P* value less than .05 provided enough confidence to reject the null (*H_0_*) hypothesis and accept the alternative hypothesis (*H_A_*). The tested categorical variable was associated with DNS events [36]. Hence, we may consider using it for future prediction.

After the data preparation process, 16 variables were selected to predict the target *dna_flag*. Among them, 12 modeling predictors were nominal variables, including binary variables (Multimedia Appendix 2). We, therefore, conducted the chi-square (*χ*^2^) statistical test to investigate the relationship between those predictors and DNS events (Table 1). The chi-square was calculated using the following equation, where O and E are observed and expected values [36,37]:



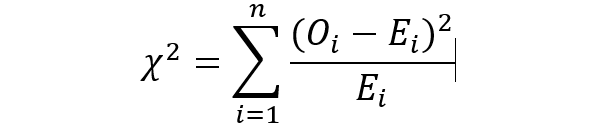



After preparing the data set and before developing the ML models, an EDA was conducted to gain a deeper understanding of the research data landscape. EDA is a fundamental data analysis required before hypothesis and modeling formulation [38]. Its findings can be used to verify misleading models at a later stage [38] and reveal unexpected patterns [39]. The EDA helped uncover patients' DNS patterns through data aggregation and data visualization analysis. Finally, the EDA findings were validated against the ML model outcomes to verify their accuracy.

**Table 1:** Chi-square test on categorical variables.

Categorical variables	Chi-square statistic	Chi-square *P* value
dna_history_count	118,461	<.01
is_last_appt_dna	77,600	<.01
Clinictypedesc	35,201	<.01
age_bins	34,810	<.01
primaryethnicityethbroadgroup3	17,098	<.01
leadtime_bins	11,048	<.01
maritalstatus_group	10,527	<.01
visit_type_group	3525	<.01
visittime_bin	3447	<.01
patcurrentdomiciledeprivationindex	2655	<.01
is_multiple_appt_same_day	1913	<.01
op_prioritycode_group	1,496	<.01
is_working_day_ind	1,244	<.01
Gender	4	.06

### Data Preprocessing

Due to the high number of categorical variables in our data set, the one-hot encoding technique was used in the preprocessing phase. Because distance-based algorithms can only deal with numerical values, in the cardinality reduction section, we used the one-hot encoding method to convert our categorical variables to numbers. After the conversion, different variables were introduced to our training data set, also known as indicator variables. For example, the variable gender derived 3 variables, *gender_male*, *gender_female*, and *gender_unknown*. Each of those variables can have a value of either 1 or 0.

As the predictive performance of classifiers is highly impacted by the selection of the hyperparameters [40], we conducted hyperparameter tuning to optimize our algorithms’ learning process. We further optimized this process using the Grid Search method to boost the performance of our chosen models. Table 2 outlines specific details regarding the hyperparameters utilized.

**Table 2:** Hyperparameter tuning of the data modeling.

Models and hyperparameters			R package		Range		Purpose
**Logistic regression**							
	penalty	Glmnet		1e-10- 1		Total amount of regularization used to prevent overfit and underfit	
**Random forest**							
	Trees	Ranger		300- 1000		Number of trees in the forest	
	Min_n	Ranger		3-10		Minimum amount of data to further split a node	
	Mtry	Ranger		3-5		Maximum number of features that will be randomly sampled to split a node	
**XGBoost^a^**							
	Trees	XGBoost		300-1000		Number of trees in the forest	
	Min_n	XGBoost		3-10		Minimum amount of data to further split a node	
	mtry	XGBoost		3-10		Maximum number of features that will be randomly sampled to split a node	
	tree_depth	XGBoost		3-12		Maximum depth of the tree	

^a^XGBoost: Extreme Gradient Boosting.

### Data Modeling

Addressing the imbalanced data set posed the main data modeling challenge. The annual DNS rate for MDHB was around 5%, which means 95% of the appointments were attended visits. This imbalance significantly affected the accuracy of our ML model in predicting attended cases. To tackle this issue, various internal and external strategies exist [41,42]. In this study, we employed an external approach that involved utilizing standard algorithms intended for a balanced data set but applying resampling techniques to the trained data set to reduce the negative impact caused by the unequal class. Our focus was on the resampling strategy, known for its effectiveness in handling imbalanced classification issues and its portability [42].

The resampling strategy involved 2 methods: (1) oversampling, where the size of the minority class is increased randomly to approach the majority class in a class-imbalance data set [43,44]; and (2) undersampling, where the size of the majority class decreases randomly to align with the minority class [43,44]. This strategy falls under both the oversampling and undersampling categories. Given the lack of definitive guidance on the effectiveness of these methods [42-44], we adopted both and compared their results.

Since we dealt with a binary classification prediction problem, supervised and classification algorithms were selected. Algorithms with good interpretability were also considered to explain which predictive variables influence DNS prediction more significantly. In a study concerning variable importance, tree-based models, such as random forest (RF) and gradient-boosted decision trees, were shown to inherently possess features that measure variable importance [45].

For the imbalanced data set, we used ensembling methods due to their proven advantages [46,47]. The following algorithms were chosen for developing DNS prediction models: logistic regression (LR), RF, and Extreme Gradient Boosting (XGBoost).

LR was chosen because it is a suitable analysis method across multiple fields for managing binary classification [48]. Our research concerned a supervised classification problem to predict whether a future outpatient appointment will become a DNS visit. With the response variable (*dna_flag*) offering dichotomous outcomes—either yes (1) or no (0)—LR stood as a fitting choice due to its proficiency in predicting binary outcomes and its established effectiveness in prior studies [7,13,33,49]. Tree-based ensembling algorithms were also chosen for their proven ability to deal with imbalanced data sets and model explainability [46,47]. RF can effectively handle combining random resampling strategies in imbalanced prediction. Tree-ensembling methods have more advanced prediction ability than a single model because they integrate prediction strength from several base learners [50].

### Model Implementation and Evaluation

We used 10-fold cross-validation for model selection and bias reduction. The hyperparameters were tuned to boost each classifier’s performance. We followed suggestions from the literature suggestions to use sensitivity, specificity, and the area under the receiver operating characteristic (AUROC) curve to quantify the models’ prediction strength for the imbalance problem prediction.

During this phase, we used the testing data to validate the best predictive model chosen based on the model evaluation criteria. For this study, data before 2021 were used in the data modeling process. We coordinated with MDHB to access outpatient appointments from 2021 for model validation. Specifically, we used both weekly and monthly data for prediction, comparing these with actual appointment outcomes to validate the model. The benefit of using a new data set for validation was to assess model bias and goodness of fit outside the research environment. Positive performance and high prediction accuracy would indicate potential real-life implementation of our research model after further investigation.

## Results

Our study only included new patients and follow-up appointments. Therefore, we analyzed DNS costs limited to new patient and follow-up outpatient services over the last 5 years. The MDHB provided us with costing information for 34 different departments, and we calculated the DNS cost for each department (Table 3). In 2020, there were 2812 new patient DNS visits and 6240 follow-up DNA visits causing a loss of at least $2.9 million (US $1.8 million) at MDHB. More information regarding this calculation is provided in Multimedia Appendix 3 [51].

Each department was assigned a corresponding outpatient appointment price for a new patient and follow-up outpatient appointment services. We aggregated the total DNS occurrences of new patients and follow-up appointments, multiplying corresponding unit prices to quantify their financial impact. For instance, in 2020, there were 301 new patients and 745 follow-up patients who missed their scheduled bookings, which caused a revenue loss of $300,442 (US $190,000) in the orthopedics department.

Although the initial research expected to address the DNS issue for all outpatient clinics and patients at the MDHB, due to the broad scope of the DNS, we concentrated on clinics with a higher percentage of DNS and narrowed down the research scope to prioritize workloads. To successfully build a model for our focused patient groups, we eliminated as many irrelevant data points as possible. Then, data used for the model training were more fit for purpose for the high-needs population.

The modeling data set was created using 649,758 records and 17 columns (Figures 1 and 3). We developed ML models based on LR, RF, and XGBoost algorithms, with hundreds of hyperparameter combinations in our data modeling. To evaluate the models’ prediction performance, accuracy, sensitivity, specificity, AUROC curves, and cost (computation time) were calculated (Table 4). The aim was to identify the best model and hyperparameters that resulted in optimal sensitivity and AUROC performances. Model prediction accuracy is critical; however, it was not a primary concern in this research as we dealt with an imbalanced data set [52].

Table 4 presents a summary comparison of the models’ performance. As shown in the table, the LR-based model was the fastest and RF the slowest in terms of computation time. LR had the lowest AUROC scores (ie, the low DNS events prediction accuracy), while RF and XGBoost had a similar area under the curve (AUC) performance (around 0.92).

**Figure 4:**
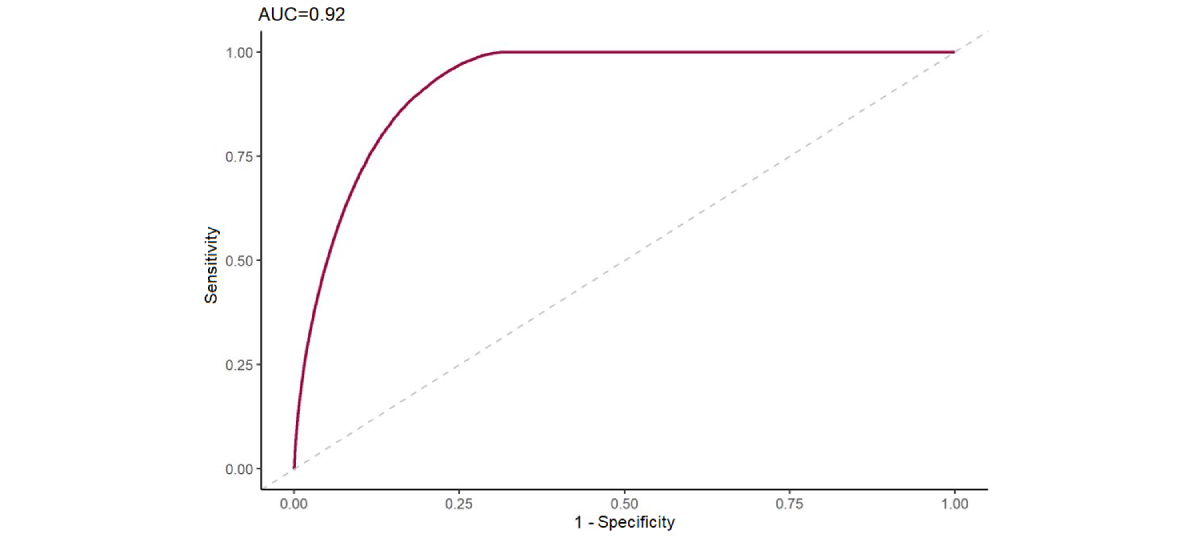
The receiver operating characteristic (ROC) of the best classifier, Extreme Gradient Boosting (XGBoost). AUC: area under curve.

The undersampling strategy significantly improved our models’ sensitivity. Sensitivity was chosen over accuracy because we were dealing with an imbalanced data set [52]. Sensitivity quantified the models’ ability to correctly predict positive (DNS) cases that help detect high-risk DNS patients. RF and XGBoost had a very close sensitivity of 0.82. However, considering the computation cost factor, XGBoost had the lowest modeling time. XGBoost with undersampling was our best ML model for the DNS prediction. Its ROC curve is illustrated in Figure 4.

A further investigation was also performed to identify the top predicting factors for each model (Multimedia Appendix 4). The purpose of calculating variable significance scores was not to plug them into a calculation formula but to showcase which variables were more relatively critical in calculating the risk of DNS. Variable importance is critical to AI model development, as variables do not contribute evenly to the final prediction. Therefore, we focused on the most influential predictors and excluded irrelevant ones by scoring the variables’ prediction contributions [53]. Variable importance is a measurement quantifying the relationship between an independent variable and the dependent [46].

The results shown in Multimedia Appendix 4 matched the chi-square statistical test results (Table 1). The leading factors were determined and selected using the variable (feature) importance. It was evident that the *dna_history_count* variable was the most influential predictor following *is_last_appt_dna*, *age_when_visit*, and *lead_time*. Additionally, *ethnicity* played an important role in constructing the XGBoost model for the DNS prediction.

We also aggregated outpatient appointment data and ranked the observed DNS rate of all outpatient clinics (Multimedia Appendix 5). We carried out this analysis to initiate an understanding of how disease type might influence the DNS rate.

**Table 3:** DNS^a^ costs in 2020 at the MDHB^b^ hospital^c^.

Clinics	NP^d^ DNS count	NP DNS price	Total NP DNS cost	FU^e^ DNS count	FU DNS price	Total FU cost	Total DNS cost
Orthopedics	301	$346	$104,143	745	$263	$196,299	$300,442
Diabetes	90	$452	$40,658	576	$307	$176,643	$217,302
Ophthalmology	221	$239	$52,776	874	$174	$152,322	$205,099
Pediatric medicine	124	$600	$74,366	327	$395	$129,271	$203,637
Ear nose throat	253	$358	$90,571	367	$269	$98,744	$189,316
Gynecology	177	$403	$71,322	386	$280	$108,124	$179,446
Hematology	75	$632	$47,389	232	$348	$80,834	$128,223
Cardiology	109	$490	$53,397	245	$299	$73,259	$126,656
Radiation oncology	42	$505	$21,194	350	$293	$102,652	$123,846
General surgery	147	$387	$56,856	208	$309	$64,369	$121,225
Audiology	268	$214	$57,302	272	$214	$58,157	$115,459
Neurology	153	$617	$94,408	38	$400	$15,204	$109,612
Gastroenterology	68	$506	$34,393	186	$362	$67,401	$101,794
Medical oncology	18	$650	$11,703	229	$360	$82,327	$94,030
Dental	136	$244	$33,132	193	$244	$47,019	$80,151
Renal medicine	5	$559	$2,793	181	$344	$62,201	$64,995
Respiratory lab	38	$479	$18,192	121	$347	$42,021	$60,213
Obstetrics	101	$227	$22,906	143	$227	$32,431	$55,337
Respiratory sleep	20	$271	$5412	153	$271	$41,403	$46,815
Urology	65	$357	$23,178	85	$274	$23,253	$46,432
Dietetics	93	$175	$16,302	168	$175	$29,449	$45,751
General medicine	44	$517	$22,747	69	$322	$22,200	$44,948
Respiratory	39	$479	$18,671	70	$347	$24,309	$42,980
Dermatology	66	$316	$20,877	60	$236	$14,174	$35,051
Oral and maxillofacial	23	$296	$6799	124	$203	$25,185	$31,984
Endocrinology	25	$525	$13,127	34	$332	$11,284	$24,411
Rheumatology	18	$647	$11,643	31	$345	$10,693	$22,336
Plastic surgery (excluding burns)	18	$296	$5321	69	$203	$14,014	$19,335
GI^f^ endoscopy	0	$506	$0	52	$362	$18,843	$18,843
Community pediatrics	20	$600	$11,994	10	$395	$3953	$15,948
Infectious diseases	7	$738	$5169	19	$534	$10,152	$15,321
Neurosurgery	1	$507	$507	29	$448	$12,990	$13,496
Podiatry	17	$207	$3522	47	$207	$9737	$13,259
Aged ATR^g^ health	18	$244	$4394	35	$244	$8545	$12,939
Under 65 ATR	3	$244	$732	5	$244	$1221	$1953
Cardiothoracic	0	$573	$0	4	$425	$1698	$1698
Anesthetics	9	0	$0	3	$0	$0	$0

^a^DNS^:^ Did Not Show.

^b^MDHB^:^ MidCentral District Health Board.

^c^A currency exchange rate of NZD $1=US $0.61 is applicable for the listed costs.

^d^NP: new patient.

^e^FU: follow-up.

^f^GI: gastrointestinal.

^g^ATR: assessment, treatment, and rehabilitation.

**Table 4:** Comparison of the ML^a^ models’ performance.

Classifier and resampling strategy		Sensitivity	Specificity	AUC^b^	Accuracy	Modeling cost
**Logistic regression**						
	Undersampling (under_ratio=2)	0.5146	0.9227	0.8474	0.8897	Less than 1 hour (5 minutes)
	Oversampling (over_ratio=0.5)	0.5091	0.9247	0.8592	0.8911	Less than 1 hour (14 minutes)
**Random forest**						
	Undersampling (under_ratio=2)	0.8243	0.8524	0.9236	0.8501	Over 8 hours (8.4)
	Oversampling (over_ratio=0.5)	0.5940	0.9260	0.9220	0.8990	Over 137 hours
**XGBoost^c^**						
	Undersampling (under_ratio=2)	0.8278	0.8490	0.9239	0.9117	Over 4 hours (4.8)
	Oversampling (over_ratio=0.5)	0.8297	0.8549	0.9267	0.8529	Over 51 hours (51.83)

^a^ML: machine learning.

^b^AUC: area under the curve.

^c^XGBoost: Extreme Gradient Boosting.

## Discussion

### Principal Findings

Our results are comparable to similar previously published analyses [9], although the AUC for XGBoost was slightly higher in our case. This may be due to the data selection and local characteristics. We initially built a generic DNS prediction model for all outpatient clinics at MDHB. However, in light of the literature and DNS complexity, the project scope was narrowed down to clinics with higher DNS rates. As discussed previously in this paper, we excluded irrelevant and missed data, invalid lead time appointments, and clinics with very low DNS rates. This approach improved the ML models' performance and made sense from an operational perspective. The developed models provided insights useful for understanding the contributing factors for DNS. We found that patient DNS history, appointment characteristics, work commitments, and socioeconomic status substantially contributed to DNS events.

### Patient DNS History

Understanding patients’ DNS history was crucial for predicting future DNS patterns (Table 5) and developing the ML models. This also aligned with the chi-square test results (Table 1), which ranked the *dna_history_count* and *is_last_appt_dna* variables as the most important factors. Total DNS counts and the latest appointment’s DNS outcome are pivotal for calculating the probabilities of future DNS occurrences. These factors are consistent with the findings in the literature [14-16,21,32,54].

Managing DNS involves identifying patients with low adherence to scheduled visits for additional attention. Centralizing and managing DNS history can provide a comprehensive view, preventing data silos or gaps. Centralized monitoring can enhance the visibility of recurring DNS incidents and proactively alert clinicians of potential DNS cases. Our models account for changes in DNS behavior. To reduce the prediction bias, we screen for the most recent appointment DNS outcome (*is_last_appt_dna*).

**Table 5:** Top prediction variables in the developed ML^a^ models.

Algorithm and variable importance ranking		Undersampling model	Oversampling model
**Logistic regression**			
	1	dna_history_count	dna_history_count
	2	is_working_day	is_working_day
	3	is_last_appt_dna	is_multiple_appt_same_day
	4	is_multiple_appt_same_day	is_last_appt_dna
	5	lead_time	lead_time
**Random forest**			
	1	dna_history_count	dna_history_count
	2	is_last_appt_dna	age_when_visit
	3	lead_time	lead_time
	4	age_bins	is_last_appt_dna
	5	clinic_type_desc	clinic_type_desc
**XGBoost^a^**			
	1	dna_history_count	dna_history_count
	2	is_last_appt_dna	age_when_visit
	3	age_when_visit	is_last_appt_dna
	4	lead_time	ethnicity
	5	Ethnicity	lead_time

^a^XGBoost: Extreme Gradient Boosting.

### Appointment Characteristics

Certain appointments expected more nonadherence, with distinct predictors related to appointment characteristics such as “working day” and “high lead time.” Longer lead times correlated with increased DNS probability, while appointments on working days were more prone to DNS than nonworking days. These findings align with reports from [33,54,55] and emphasize the significant impact of appointment lead time on DNS prediction, as also indicated in [8,14,16,32,33,54]. This underscores how appointment characteristics directly affect DNS outcomes immediately after scheduling. Therefore, incorporating ML-predicted DNS risk estimations during appointment scheduling could automatically flag higher DNS probability for proactive management.

Furthermore, our analysis of the *op_prioritycode* variable (Multimedia Appendix 1) indicated that, in general, patients with more serious health conditions were more likely to attend their appointments. This observation is reflected in Multimedia Appendix 5, which compares the DNS rates of different clinics with the overall average DNS rate of 0.053% (depicted red line). For example, patients visiting the audiology clinic had a potential DNS rate of 19.1% compared to a 0.9% DNS rate for the radiation oncology clinic. Our analysis of the *op_prioritycode* variable was based on categorical data types reflecting appointment urgency and not based on a detailed analysis of each patient’s diagnosis.

### Work Commitments

Our findings suggest that patients struggled to adhere to appointments on working days or during working hours. Younger adults, particularly those between 20 and 30 years of age, had higher DNS rates due to work commitments, while older adults aged 65 years and above rarely missed their visits.

Furthermore, the XGBoost-based model highlighted that being single was an indicator of DNS visits (Figure 4). This could relate to time constraints among young professionals, a finding consistent with other studies [8,28,33,56]. For this group, a targeted reminder system could be developed to concentrate on appointments with higher DNA probability compared to the DNS risk threshold. Consequently, the population-based reminding system could help optimize resource allocation, including staff efforts and costs.

### Socioeconomic Status

We explored the deprivation index and clustered patient populations by using their ethnicity (Multimedia Appendix 6). Our findings indicated a strong association between European and Māori ethnicities and DNS outcomes, ranked among the top 5 predicting factors (Multimedia Appendix 4). Māori and Pacific populations had the highest DNS rates, in line with other research findings [56], while the European ethnicity had the lowest DNS rates. Māori and Pacific populations tended to reside in areas characterized by higher deprivation rates, whereas the percentage of other ethnicities living in higher deprivation regions decreased when the deprivation index increased.

In New Zealand, Māori and Pacific ethnical groups required increased health care attention [57] to ensure equity in the health care system. As indicated in Table 6, a larger proportion of these ethnic groups are situated in suburbs and areas with higher deprivation indexes (such as 8, 9, and 10) [58]. The higher deprivation index was also a strong indicator of socioeconomic deprivation geographically [58]. According to the New Zealand Index of Deprivation, neighborhoods with higher deprivation were more likely to experience adverse living conditions such as damp, cold, and crowded housing.

Moreover, regions with higher deprivation exhibit higher rates of unemployment, increased dependence on benefits, and more single-parent families [58]. Consequently, these living conditions and income disparities made patients living in these regions more susceptible to illness, while also encountering more barriers and obstacles in addressing their medical needs.

At MDHB, dedicated working groups were established to support Māori and Pacific patients in attending their scheduled hospital appointments. Our research reiterates the importance and necessity of those working groups, acknowledging the value of their work. Moreover, our model can support them further by providing tangible DNS probability scores to prioritize patients who require additional attention and support.

**Table 6:** Percentage of population residing at each deprivation level [58].

Deprivation level	Māori, n (%)	Pacific, n (%)	European, n (%)	Asian, n (%)	Other, n (%)
1	3113 (7)	293 (1)	37,314 (86)	2077 (5)	835 (2)
2	4951 (9)	429 (1)	46,405 (85)	1470 (3)	1071 (2)
3	6367 (13)	489 (1)	42,565 (84)	613 (1)	821 (2)
4	14,736 (14)	1747 (2)	84,728 (79)	4574 (4)	1593 (1)
5	14,400 (13)	3398 (3)	83,568 (77)	6015 (6)	1590 (1)
6	14,103 (15)	1759 (2)	74,351 (79)	2974 (3)	1248 (1)
7	13,442 (17)	3601 (5)	58,187 (75)	1858 (2)	870 (1)
8	36,843 (19)	5402 (3)	148,605 (75)	5434 (3)	1988 (1)
9	40,642 (24)	7324 (4)	111,319 (67)	5443 (3)	2442 (1)
10	31,998 (35)	6283 (7)	52,064 (56)	1610 (2)	521 (1)

### Operational and Managerial Implications

The total DNS loss incurred by the MDHB hospital was around $2.9 million (US $1.8 million) in 2020. Notably, we observed that clinics with less life-threatening diseases (diabetes, audiology, and dental) had higher DNS rates. Considering our use of MDHB data, we expect to identify similar patterns in other district health boards for which the same DNS predicting factors can be applied for DNS management.

While the primary objective of our research was to calculate DNS risk for promoting health equity, we believe that leveraging DNS prediction can aid in managing limited health care resources more efficiently. By quantifying the DNS probability for future appointments on a scale from 0.00 to 1, clinicians or hospital operation managers can develop more personalized health care services for their patients. This leads to enhancing equity in accessing health care services for a wider population.

The predictions derived can support MDHB managers in designing, planning, and implementing more informed DNS management strategies. For example, a DNS appointments threshold (eg, 0.7) can be set, and all appointments with predicted odds greater than 0.7 can be selected, releasing 70% of resources and allocating some (or all) to the remaining 30% of patients with a higher DNS risk. Potentially, these released resources can subsidize interventions to support attendance. Without DNS prediction, the hospital cannot decide where to focus on solving the DNS problem and must invest money uniformly for every patient, leading to equality rather than equity in health care service access. Equality is not fit for purpose, especially considering the high attendance rate of 95% over the past 5 years, indicating that most patients attend appointments without additional support. However, for more optimum use of health care resources, other policies and guidance for appointment scheduling should be considered [59].

### Potential Interventions to Reduce DNS

#### DNS Suggests Life Hardships

When patients miss medical appointments, it is a critical indicator suggesting they may be experiencing hardships in their lives [15,54,60]. Considering that a higher DNS rate correlates with a higher deprivation index, we can assume that people residing in these areas may face greater transportation limitations. Moreover, people with severe mental health or addiction issues may not be able to independently visit their doctors [15]. These vulnerable groups require additional and ongoing appointment assistance. Unfortunately, they have been historically disadvantaged and marginalized by the current health care system [61].

The DNS prediction model we developed can help health care practitioners identify patients at higher risk of DNS. Targeted DNS improvement solutions can be designed based on predicted DNS probability, patient demography, and clinical history. This type of application can leverage the DNS prediction model to help identify and deliver patient-centric medical services to patients requiring additional help. Some examples are discussed in the subsequent sections.

#### Expanding Integrated Health Care Networks

For patients not facing life-threatening illnesses or requiring long-term health management (such as patients with diabetes), expanding services closer to patients might help meet their needs. MDHB could consider deploying clinicians to outsourced sites to supervise practitioners or attend to patients directly. Moreover, increasing collaborations with primary health care networks, promoting nurse-led services, and contracting private specialists can also be viable options for decreasing DNS rates. Developing a one-stop medical hub with multidisciplinary clinics for patients with lower clinical risk could encourage attendance and reduce DNS visits [19]. This is consistent with the New Zealand Ministry's latest health care system reform strategies, which aim to uplift health care equity [61]. The reform emphasizes the establishment of more locality networks in the community, resonating well with our research findings.

#### After-Hour Appointment Slots

To support young adults who are occupied by daily work, it might be favorable to increase more after-hour service slots in clinics when possible. If more appointment slots can be organized before or after working hours, working professionals may have more chances to adhere to their clinical appointments. Piloting more weekend clinics can also be a choice to meet younger generations’ needs. In consonance with our suggestion, the recent New Zealand health care reform also promoted more affordable after-hours services [61]. Additionally, offering transportation assistance and improved wraparound well-being support for patients with a high-risk score could increase attendance. At-home patient visits could also be offered and delivered to patients facing severe transport limitations.

### Limitations

Despite the success of our DNS prediction model, we need to acknowledge that it has some limitations. First, our model was trained on 5-year period data from MDHB. The single data source prevented us from exploring other critical dimensions such as household data or beneficiary data. We believe adding those data points would improve the prediction model and discover more patients’ DNS patterns.

Furthermore, we pairwise compared the attribute *dna_flag* with other DNS predictor factors. However, future research should consider investigating and analyzing the association between variables and adding further variables to the conditioning set. This expanded analysis would offer deeper insights into patients' DNS behaviors.

### Conclusions

To the best of our knowledge, this study represents one of the first attempts in New Zealand to develop ML prediction models supporting DNS management. We successfully developed and tested ML models to predict probabilities of outpatient appointments’ DNS. Our selected model had an AUROC of 0.92 and a sensitivity performance of 0.82. 

## Appendix

Multimedia Appendix 1 Details of variables and their definitions.

Multimedia Appendix 2 Data type of original and newly derived variables.

Multimedia Appendix 3 Outpatient appointment prices.

Multimedia Appendix 4 Leading predicting factors of the best Extreme Gradient Boosting (XGBoost) model.

Multimedia Appendix 5 Did Not Show (DNS) rates of all outpatient clinics of the MidCentral District Health Board (MDHB) hospital.

Multimedia Appendix 6 Did Not Show (DNS) rates among different deprivation groups and ethnicities.

## Abbreviations

AI: artificial intelligence
AUC: area under the curve
AUROC: area under the receiver operating characteristic
AUT: Auckland University of Technology
DNA: Did Not Attend
DNS: Did Not Show
EDA: exploratory data analysis
FTA: Failed To Attend
LR: logistic regression
MDHB: MidCentral District Health Board
ML: machine learning
RF: random forest
ROC: receiver operating characteristic
XGBoost: Extreme Gradient Boosting
