# Supplementary material for: Enhancing Health Equity by Predicting Missed Appointments in Health Care: Machine Learning Study
Source: JMIR Med Inform. 2024 Jan 12;12:e48273. doi: 10.2196/48273 (PMC10818230; doi:10.2196/48273)
Supplement: Multimedia Appendix 1 [file medinform_v12i1e48273_app1.docx]

## Multimedia Appendix 1: Details of variables and their definitions

| Variable name | Variable Type | Variable Definition |
| --- | --- | --- |
|  |  |  |
| **dna_flag** |  |  |
|  | logical (0,1) | whether an appointment was a dna visit or not. ‘1’ means a did-not-attend visit, and ‘0’ means attended visit |
|  |  |  |
| **visitdatetime** |  |  |
|  | datetime | date and time format of an appointment |
|  |  |  |
| **Date variables (4)** |  |  |
|  | logical (0,1) | whether an appointment was booked on a working day or non-working day (either weekend or public holiday) |
|  |  |  |
| **bookingentrydatetime** |  |  |
|  | datetime | when an appointment was booked |
|  |  |  |
| **last_appt_date** |  |  |
|  | date | when is the appointment date of a referral (one referral can have multiple appointments), if any |
|  |  |  |
| **clinictypedesc** |  |  |
|  | categorical | description of clinic type (such as Mc Diabetes, Mc Audiology, and so on) |
|  |  |  |
| **op_prioritycode** |  |  |
|  | categorical | priority code of an appointment which indicates how urgent an appointment was |
|  |  |  |
| **visittypecode** |  |  |
|  | categorical | type of an appointment (such as new patient, follow-up, and so on) |
|  |  |  |
| **Visittypelongdesc** |  |  |
|  | categorical | full description of the visittypecode |
|  |  |  |
| **unique_id** |  |  |
|  | categorical | encrypted code of patient National Health Index (NHI)  number |
|  |  |  |
| **age_when_visit** |  |  |
|  | integer | patient age as of the appointment date |
|  |  |  |
| **patcurrentdomiciledeprivationindex** |  |  |
|  | categorical | current patient deprivation index (from 1 to 10) |
|  |  |  |
| **primaryethnicityethbroadgroup3** |  |  |
|  | categorical | patient ethnicity code (based on the level 3 code of the ministry standard) |
|  |  |  |
| **gender** |  |  |
|  | categorical | patient gender (m-male, f-female, u-unknown) |
|  |  |  |
| **maritalstatus** |  |  |
|  | categorical | current marital status of patients |
|  |  |  |
| **referralno** |  |  |
|  | categorical | referral number to which an appointment attached |
|  |  |  |
| **prioritydate** |  |  |
|  | date | when an appointment was prioritised (triaged) |
|  |  |  |
