# Supplementary material for: Enhancing Health Equity by Predicting Missed Appointments in Health Care: Machine Learning Study
Source: JMIR Med Inform. 2024 Jan 12;12:e48273. doi: 10.2196/48273 (PMC10818230; doi:10.2196/48273)
Supplement: Multimedia Appendix 2 [file medinform_v12i1e48273_app2.docx]

## Multimedia Appendix 2: Data type of original and newly derived variables

Table B. Data types and variables

| Variable type | Modelling variables (17) | Redundant variables (10) |
| --- | --- | --- |
|  |  |  |
| **Nominal variables (13)** |  |  |
|  | clinictypedesc, op_prioritycode, visittypecode, patcurrentdomiciledeprivationindex, primaryethnicityethbroadgroup3, gender, maritalstatus, visittime_bin, age_bins, leadtime_bins | visittypelongdesc, unique_id, referralno |
|  |  |  |
| **Binary variables (4)** |  |  |
|  | dna_flag, is_working_day_ind, is_multiple_appt_same_day, is_last_appt_dna |  |
|  |  |  |
| **Date variables (4)** |  | last_appt_date, prioritydate, booking_date, visitdate |
|  |  |  |
|  |  |  |
| **Datetime variables (2)** |  |  |
|  |  | visitdatetime, bookingentrydatetime |
|  |  |  |
| **Time variable (1)** |  |  |
|  |  | visittime |
|  |  |  |
| **Integer variables (3)** |  |  |
|  | age_when_visit, lead_time, dna_history_count |  |
|  |  |  |
