# Supplementary material for: Enhancing Health Equity by Predicting Missed Appointments in Health Care: Machine Learning Study
Source: JMIR Med Inform. 2024 Jan 12;12:e48273. doi: 10.2196/48273 (PMC10818230; doi:10.2196/48273)
Supplement: Multimedia Appendix 3 [file medinform_v12i1e48273_app3.docx]

## Multimedia Appendix 3: Outpatient appointments prices

In addition to the research data set, the MDHB hospital supplied a supplementary

data set including the national standard Purchase Unit Code (PUC) and corresponding cost of all its outpatient services. According to New Zealand Ministry of Health, the PUC code is a standardised and 95 consistent classification system for service measurement, quantification, and value estimation [60]. The PUC system was reviewed each year to retire old clinical services and introduce new ones satisfying patient needs. As discussed in the data exclusion part, we only included new patient and follow-up appointments for this research. Therefore, we filtered out PUC information limited to new-patient (NP) and follow-up (FU) outpatient services of the last five years from the given PUC data set. The data set used for the costing analysis had four columns consisting of cdFiscalYear, cdPUCCode, cdMeasure, cdPrice, and pucDescription. Overall, 34 different departments were assigned a corresponding PUC code and price to a new patient and follow-up outpatient appointment services. A subset of 2020 PUC prices of the NP and FU outpatient services is listed in table A.

Table A. Outpatient appointments prices of the MDHB in 2020 (all prices are in New Zealand Dollar)

| Department | New Patient (NP) DNS Unit Price | Follow Up (FU) DNS Unit Price |
| --- | --- | --- |
|  |  |  |
| **Infectious Diseases** | 738.38 | 534.31 |
|  |  |  |
| **Pain Clinic** | 704.61 | 383.56 |
|  |  |  |
| **Medical Oncology** | 650.18 | 359.51 |
|  |  |  |
| **Rheumatology (including immunology)** | 646.84 | 344.94 |
|  |  |  |
| **Haematology** | 631.85 | 348.42 |
|  |  |  |
| **Neurology** | 617.05 | 400.11 |
|  |  |  |
| **Paediatric Medical Outpatient** | 599.72 | 395.32 |
|  |  |  |
| **Cardiothoracic Surgery** | 572.72 | 424.62 |
|  |  |  |
| **Renal Medicine** | 558.69 | 343.65 |
|  |  |  |
| **Endocrinology** | 525.08 | 331.88 |
|  |  |  |
| **General Medicine** | 516.98 | 321.75 |
|  |  |  |
| **Neurosurgery** | 506.52 | 447.93 |
|  |  |  |
| **Gastroenterology** | 505.78 | 362.37 |
|  |  |  |
| **Radiation Oncology** | 504.62 | 293.29 |
|  |  |  |
| **Cardiology** | 489.88 | 299.02 |
|  |  |  |
| **Respiratory** | 478.74 | 347.28 |
|  |  |  |
| **Diabetes** | 451.76 | 306.67 |
|  |  |  |
| **Gynaecology** | 402.95 | 280.11 |
|  |  |  |
| **General Surgery** | 386.78 | 309.47 |
|  |  |  |
| **Ear Nose Throat** | 357.99 | 269.06 |
|  |  |  |
| **Urology** | 356.59 | 273.57 |
|  |  |  |
| **Orthopaedics** | 345.99 | 263.49 |
|  |  |  |
| **Dermatology** | 316.32 | 236.23 |
|  |  |  |
| **Plastic Surg. (including Burns and Maxillofacial)** | 295.61 | 203.10 |
|  |  |  |
| **Respiratory Sleep** | 270.61 | 270.61 |
|  |  |  |
| **ATR Outpatient** | 244.13 | 244.13 |
|  |  |  |
| **Dental** | 243.62 | 246.62 |
|  |  |  |
| **Ophthalmology** | 238.81 | 174.28 |
|  |  |  |
| **Fracture Clinic** | 238.63 | 299.77 |
|  |  |  |
| **Obstetrics** | 226.79 | 226.79 |
|  |  |  |
| **Audiology** | 213.81 | 213.81 |
|  |  |  |
| **Podiatry** | 207.18 | 207.18 |
|  |  |  |
| **Dietetics** | 175.29 | 175.29 |
|  |  |  |
| **Anaesthetics** | 0.00 | 0.00 |
