# Supplementary figures and images for: Enhancing Health Equity by Predicting Missed Appointments in Health Care: Machine Learning Study
Source: JMIR Med Inform. 2024 Jan 12;12:e48273. doi: 10.2196/48273 (PMC10818230; doi:10.2196/48273)

## Multimedia Appendix 4: Leading predicting factors of the best XGBoost model


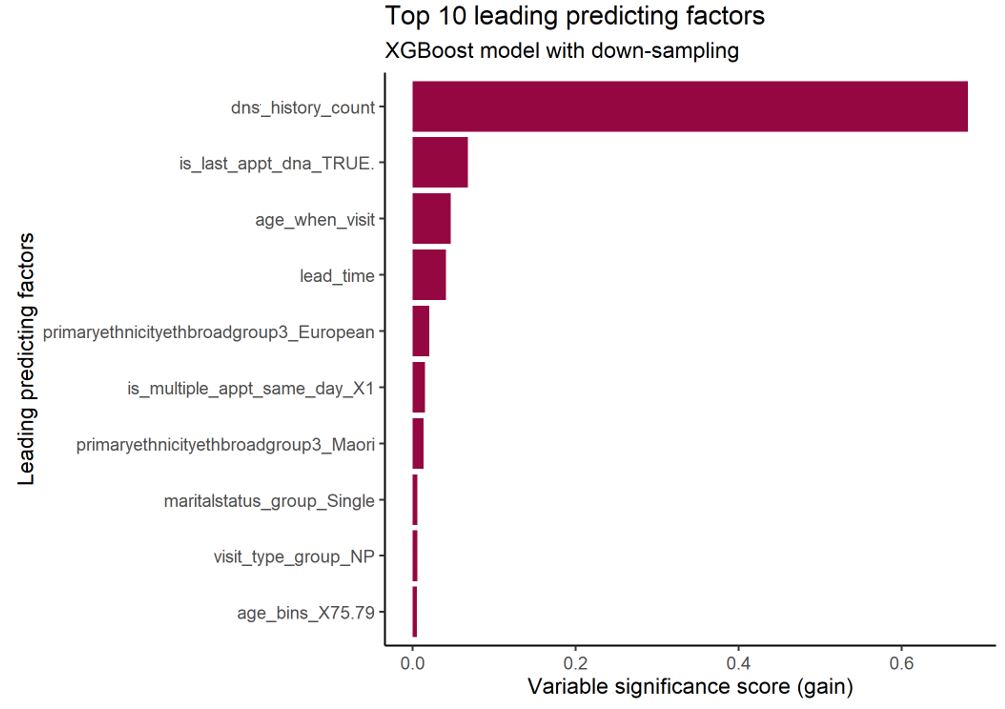

Supplement: Multimedia Appendix 4 [file medinform_v12i1e48273_app4.docx]

## Multimedia Appendix 5: DNS rates of all outpatient clinics of the MDHB hospital


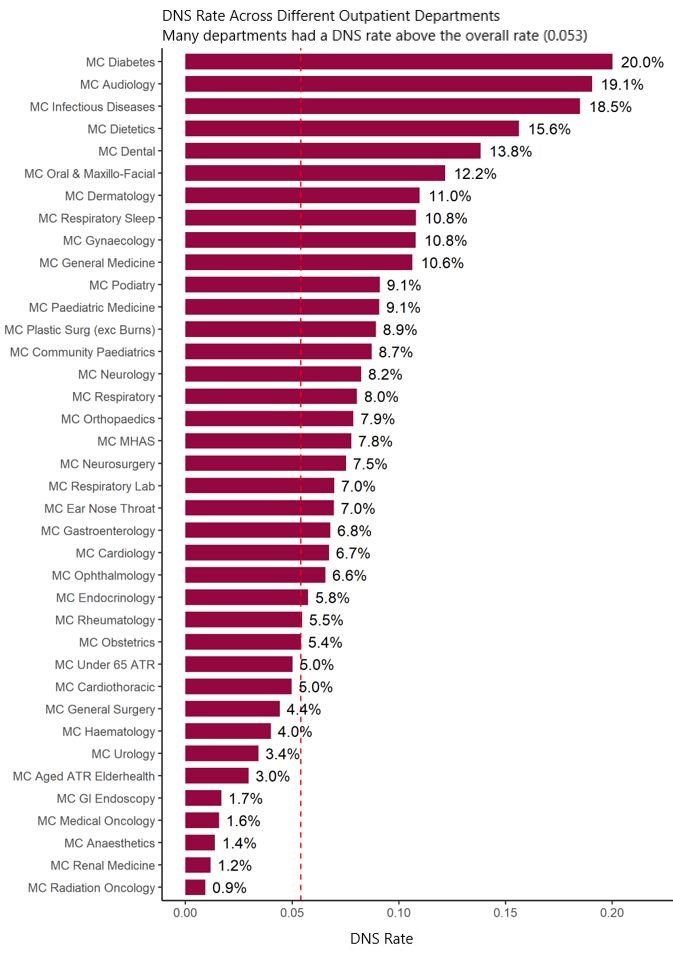

Supplement: Multimedia Appendix 5 [file medinform_v12i1e48273_app5.docx]

## Multimedia Appendix 5: DNS in different deprivation groups and ethnicities


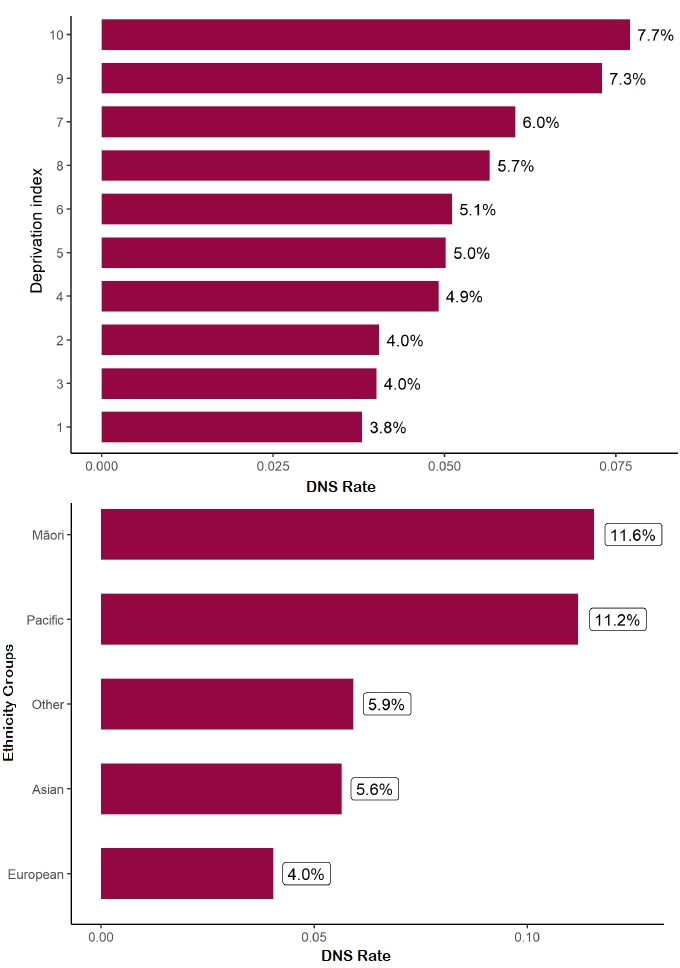

Supplement: Multimedia Appendix 6 [file medinform_v12i1e48273_app6.docx]
